# A Multi-Individual Pharmacokinetic Model Framework for Interpreting Time Trends of Persistent Chemicals in Human Populations: Application to a Postban Situation

**DOI:** 10.1289/ehp.0900648

**Published:** 2009-05-01

**Authors:** Roland Ritter, Martin Scheringer, Matthew MacLeod, Urs Schenker, Konrad Hungerbühler

**Affiliations:** Safety and Environmental Technology Group, ETH Zurich, Zurich, Switzerland

**Keywords:** biomonitoring, DDT, exposure science, modeling, persistent organic pollutants

## Abstract

**Background:**

Human milk and blood are monitored to detect time trends of persistent organic pollutants (POPs) in humans. It is current practice to use log-linear regression to fit time series of averaged cross-sectional biomonitoring data, here referred to as cross-sectional trend data (CSTD).

**Objective:**

The goals of our study are to clarify the interpretation of half-lives derived from fitting exponential functions to declining CSTD and to provide a method of estimating human elimination half-lives from CSTD collected in a postban situation.

**Methods:**

We developed a multi-individual pharmacokinetic model framework and present analytical solutions for a postban period. For this case, the framework quantitatively describes the relationships among the half-life for reduction of body burdens of POPs derived from CSTD, the half-life describing decline in daily intake, and the half-life of elimination from the human body.

**Results:**

The half-life derived from exponential fitting of CSTD collected under postban conditions describes the exposure trend and is independent of human elimination kinetics. We use a case study of DDT (dichlorodiphenyltrichloroethane) to show that CSTD can be combined with exposure data obtained from total diet studies to estimate elimination kinetics of POPs for humans under background exposure conditions.

**Conclusions:**

CSTD provide quantitative information about trends in human exposure and can be combined with exposure studies to estimate elimination kinetics. The full utility of these data has not been exploited so far. An efficient and informative monitoring strategy for banned POPs in humans would coordinate sampling of consistent sets of CSTD from young adults with total diet studies.

The Stockholm Convention on Persistent Organic Pollutants (POPs) entered into force in 2004 and mandates restriction and eventual elimination of selected chemicals at an international level to protect human health and the environment [United Nations Environment Programme (UNEP) 2009]. Article 16 of the convention requires an effectiveness evaluation of measures taken to reduce emissions of POPs. To this end, a global monitoring plan is currently being developed with the goal of detecting trends in levels of POPs in air, human milk, and human blood over time (UNEP 2007). The prospective character of this global monitoring plan will make it possible to control variables such as age and, for females, parity of donor individuals.

So far, long-term trends of POPs in humans have often been evaluated retrospectively by collecting several sets of cross-sectional data (CSD), each describing levels in human tissue in different individuals at one point in time. Summary statistics such as arithmetic means and medians of CSD obtained at different times are used to assemble time series from which a trend can be derived. Here, we refer to time trends derived from CSD as “cross-sectional trend data” (CSTD). Declining trends in CSTD typically occur after use and production of a chemical has been restricted or banned, and are often fitted with exponential functions (Craan and Haines 1998; Erdogrul et al. 2004; Jaraczewska et al. 2006; Minh et al. 2004; Noegrohati et al. 1992; Norén and Meironyté 2000; Smith 1999). Exponential fitting, that is, log-linear regression, is also a method proposed in the global monitoring plan (UNEP 2007).

Presently, no consistent nomenclature exists to describe this CSTD-based half-life or its corresponding rate constant. Noegrohati et al. (1992) used the expression “disappearance rate constant,” Norén and Meironyté (2000) used the term “decline half-time,” Sjödin et al. (2004) used “populational half-life,” and Smith (1999) used “population level half-life” to distinguish it from the human elimination half-life. CSTD-derived half-lives have also been referred to as “human half-life” (Woodruff et al. 1994), “half-life in humans” (Al-Saleh et al. 2003; Ennaceur et al. 2007; Solomon and Weiss 2002; Wolff 1999), and “half-life in the human body” (Cocco et al. 2000).

Two factors have been reported in the literature as influencing the CSTD-based half-life. The first is the trend of exposure or intake. It is intuitively apparent that a declining trend in exposure will result in a declining trend in CSTD. Accordingly, CSTD studies of human blood or milk are used as markers of exposure and to track reductions in exposure (UNEP 2007; World Health Organization 2007). The second factor that has been reported to influence CSTD-based half-lives is the rate of elimination of a substance from the body by all possible pathways, which we refer to as the “human elimination half-life.” Currently, however, the influence of exposure trend and elimination kinetics on CSTD-derived trends is unclear, and a range of different interpretations exists in the literature. For instance, the CSTD-based half-life has been interpreted to reflect both elimination kinetics and intake trend (Sjödin et al. 2004), to be consistent with the half-life derived from sequential measurements in individuals (Wolff 1999), or to be confounded by ongoing exposure (Minh et al. 2004). Both a single-individual pharmacokinetic (PK) model (Aylward and Hays 2002) and a multi-individual PK model (Lorber 2002) have been applied to describe the trend observed in CSTD. This variability in interpretations reflects the lack of a mechanistic description of the CSTD-based half-life and the lack of standard nomenclature.

In contrast to CSTD, which represent the population level, the role of the time trend of intake and of elimination kinetics is well established for single individuals. Concentration trends in individuals, which are referred to as “longitudinal data” (LD) (Tee et al. 2003), have been mechanistically described by single-individual PK models of different complexity (van der Molen et al. 1996; Verner et al. 2008). In LD, the trend in body burden is determined by both the intake trend and elimination kinetics. As a consequence, background intake is a possible confounding factor in estimates of the human elimination half-life from LD when complete isolation from ongoing exposure is not feasible (Shirai and Kissel 1996).

The present study has two major goals. The first is to clarify the interpretation of half-lives derived from CSTD fitted with an exponential function by using a transparent and mathematically explicit model of the relationships among *a*) CSTD-based half-life, *b*) time trend of intake, and *c*) the human elimination half-life, in a postban period. The second goal is to provide a new approach to estimating human elimination half-lives; this approach uses CSTD that represent the adult population under background exposure conditions in combination with exposure studies.

## Methods

**Figure 1:**
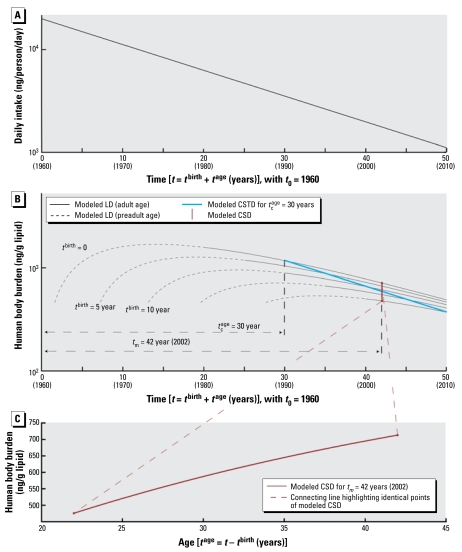
Schematic overview of the model framework. (*A*) Background daily intake, *I*(*t*) (Equation 1). (*B*) Modeled LD (Equation 6), representing the time trend of single individuals, modeled CSTD (Equation 7), and modeled CSD as functions of time. (*C*) Modeled CSD from (*B*) shown as a function of age (Equation 9).

To derive our multi-individual PK framework, we first formulated a one-compartment PK model for a single individual with a time-dependent intake function, *I*(*t*), which is assumed to decline exponentially in a post-ban phase with a first-order rate constant *k*_dec_ (years^−1^):


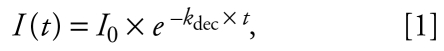


where *t* (years) is time (starting at *t*_0_, where *t*_0_ = 0 in the following equations), and *I*_0_ is the intake (nanograms per person per day) at *t*_0_. This intake function is shown in Figure 1A. Other intake functions can also be used if the model is solved numerically [see Supplemental Material (doi:10.1289/ehp.0900648.S1)].

In most cases, human exposure to POPs occurs mainly through dietary intake (Darnerud et al. 2006). In this context, we use the term “intake” as synonymous with background exposure. The one-compartment PK model for an individual born at *t*_0_ is represented by the following first-order differential equation:


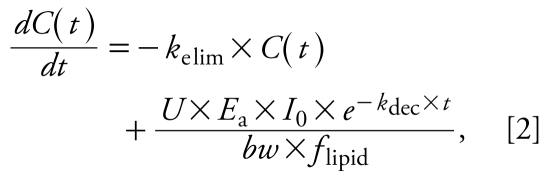


where *C*(*t*) is the concentration (nanograms per gram lipid) in the body on a lipid-normalized basis (also referred to as body burden) as a function of time, *k*_elim_ (years^−1^) is the first-order rate constant for elimination of contaminant from the human body, *bw* is body weight in kilo grams, *f*_lipid_ (dimensionless) is the lipid fraction of the human body, and *U* is a unit conversion factor (days × year^−1^ × kg × g^−1^). *E*_a_ (dimensionless) describes the fraction of chemical absorbed (i.e., intake × *E*_a_ = uptake) and is set at 0.9 (Moser and McLachlan 2001). In some contexts, it is more common to use a half-life rather than a rate constant. The human elimination half-life follows from the rate constant as *t*_1/2_^elim^ (years) = ln(2)/*k*_elim_. Analogously, *t*_1/2_^dec^ (years) = ln(2)/*k*_dec_ denotes the half-life describing the decline of intake. Integrating Equation 2 with boundary condition *C*(*t*_0_) = 0 yields a function describing the body burden as a function of time for an individual born at *t*_0_ for *k*_dec_ ≠ *k*_elim_:


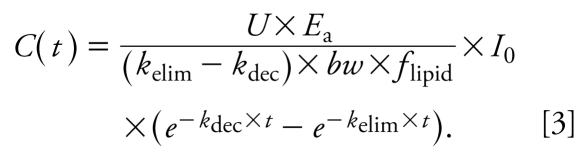


[For the derivation of this solution and the solution for the special case *k*_dec_ = *k*_elim_, see Supplemental Material (doi:10.1289/ehp.0900648.S1).] This equation can be generalized to a family of equations for individuals born at *t*^birth^ ≠ *t*_0_ by, first, substituting *I*_0_, the intake at time *t*_0_, by *I*_birth_, the intake at the time of birth of each individual:


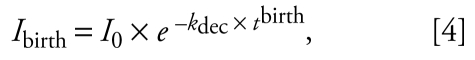


and second, introducing a variable *t*^age^ that describes the age of an individual born at *t*^birth^ and is given by *t*^age^ = *t* − *t*^birth^. The function *C*(*t*) from Equation 3 is then generalized to a function in two variables, *t*^birth^ and *t*^age^:


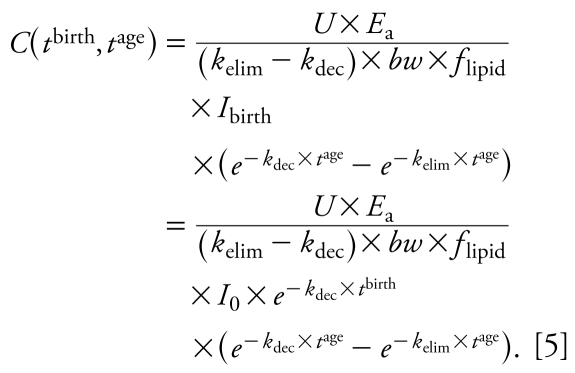


**Figure 2:**
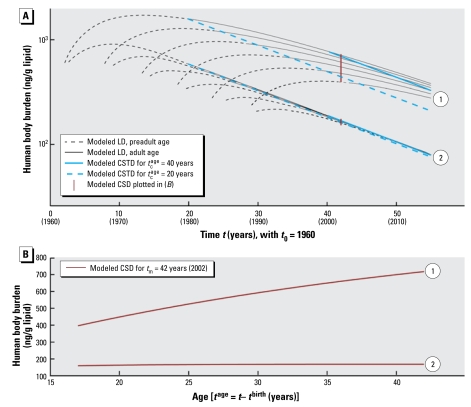
Influence of human elimination half-life, *t*_1/2_^elim^, and characteristic age, *t*_c_^age^, on modeled CSTD and CSD. We assume an initial daily intake, *I*_0_, of 20,000 ng/person/day for two chemicals: chemical 1, with *t*_1/2_^elim^ = 8 years, and chemical 2, with *t*_1/2_^elim^ = 3 years; the half-life of decline in exposure, *t*_1/2_^dec^, is the same for both chemicals and is assumed to be 12 years. (*A*) For the slowly eliminated chemical 1, concentrations in humans are higher than for the more rapidly eliminated chemical 2. In addition, the trend lines for two characteristic ages, 20 years and 40 years, are separated for chemical 1 but are almost identical for chemical 2. However, all four CSTD lines are parallel and reflect, according to Equation 7, the half-life in decline of intake, *t*_1/2_^dec^, of 12 years. “Preadult” indicates < 20 years of age. (*B*) Modeled CSD, calculated for the year 2002, as a function of age. Only the slowly eliminated chemical 1 shows a significant increase of concentration with age.

**Figure 3:**
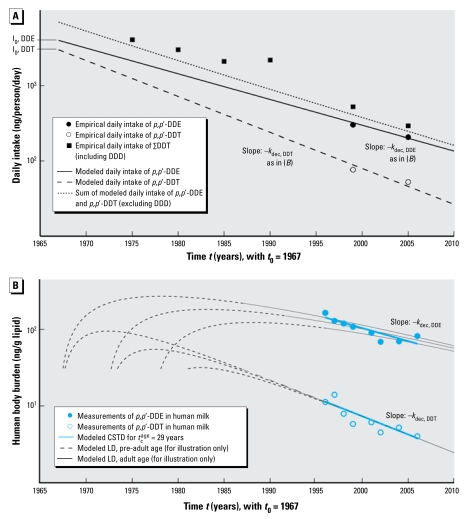
Illustration of the five-step procedure for estimating human elimination half-lives: results for *p*,*p*′-DDE and *p*,*p*′-DDT are based on empirical data from Sweden (Ankarberg et al. 2006; Darnerud et al. 2006; Glynn et al. 2007). (*A*) Modeled and empirical daily intakes used in steps 3 and 4 of the procedure. (*B*) Modeled and empirical CSTD used for steps 1, 2, and 5 of the procedure. Each circle represents the average of one set of CSD. See “Model application: a case study of DDT (dichlorodiphenyltrichloroethane)” for a detailed description of the five-step procedure. For additional information, see Supplemental Material, Tables S1 and S2 (doi:10.1289/ehp.0900648.S1).

This general equation can be used to explain three types of concentration–time relationships. The first is the time course of LD for an individual that enters the population in year *t*_c_^birth^. Here, we set *t*^birth^ at a constant value, *t*_c_^birth^, and consider only *t*^age^ as a variable (Figure 1B):


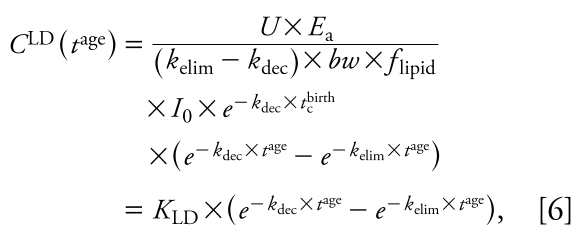


with *K*_LD_ as a constant independent of *t*^age^. Equation 6 is analogous to one-compartment PK equations describing the time course of concentration in the body over hours or days after drug administration in an individual. However, in the long-term context applied here (years), Equation 6 describes the body burden over the lifetime of an individual that enters the world as an adult at *t* = *t*_c_^birth^. In reality, early life years are characterized by important physiologic changes (e.g., growth) and changes in intake (e.g., end of breast-feeding). Therefore, the concentration course shown for early life years as dashed lines in Figures 1–3 should not be interpreted as estimates of actual concentration–time courses; these parts of the curves are plotted for illustrative purposes only.

The second relationship derived from Equation 5 is the concentration time course observed in CSTD. CSTD represent a time trend in data taken from groups of individuals at different times. Each data point in the time trend represents the arithmetic mean or median of measurements in several individuals; that is, each of the data points in a set of CSTD is representative of the cohort sampled in the underlying cross-sectional study. To be comparable, all CSD sets that are used to build the CSTD should represent a population with the same or at least similar characteristics such as age, body weight, and parity. We refer to empirical CSTD that have been derived from similar CSD sets as “consistent CSTD.” We define *t*_c_^age^ as the constant “characteristic age” that is representative of the cohorts measured in all CSD sets included in a set of consistent CSTD. In our framework, each set of CSD is modeled as an average individual representing a specific birth cohort born at *t*^birth^ and measured at age *t*_c_^age^. With constant *t*_c_^age^ we can derive from Equation 5 an explicit expression for CSTD as function of *t*^birth^:


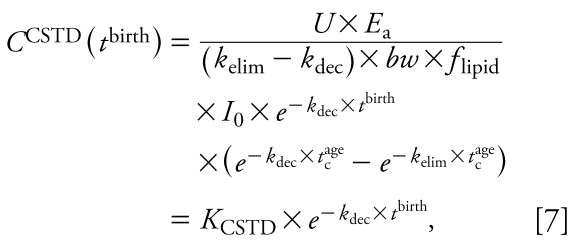


with *K*_CSTD_ as a constant independent of *t*^birth^. Equation 7 is illustrated in Figure 1B.

Third and finally, Equation 5 can be used to derive an explicit relationship between concentration and age as measured in CSD at a specified time. If we recall that *t* = *t*^birth^ + *t*^age^ and, thus, *t*^birth^ = *t* − *t*^age^, replacing *t*^birth^ by *t* − *t*^age^ in Equation 5 yields


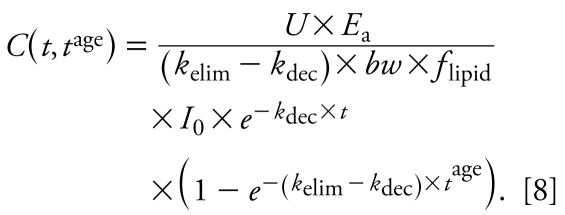


In a set of CSD, the time *t* at which the CSD were measured is constant, here denoted by *t*_m_. Equation 8 can then be simplified to an expression for concentration as function of age:


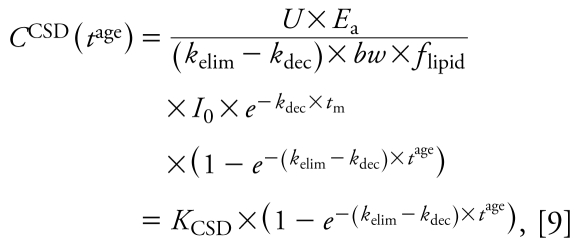


with *K*_CSD_ as a constant independent of *t*^age^. Equation 9 is illustrated in Figure 1C.

## Results

### Properties of modeled CSTD

Equation 7 provides two new insights into the exponential decay function that has been fitted to CSTD in numerous publications. First, Equation 7 demonstrates that in a postban situation the half-life derived from exponential fitting of CSTD is equal to the half-life of decline in intake, *t*_1/2_^dec^, and is completely independent of human elimination kinetics. Second, the initial value of the exponential function, *K*_CSTD_, is now mechanistically characterized, which reveals the influence of specific PK parameters on CSTD. These two insights provide the basis for a new methodology to estimate a human elimination half-life, *t*_1/2_^elim^, that is representative of the general adult population under background exposure conditions.

Figure 2A illustrates the influence of changes in *k*_elim_ and *t*_c_^age^ on modeled CSTD. A change in *k*_elim_ causes a parallel shift of the logarithmic CSTD function. As can be deduced from Equation 7, other parameters such as *I*_0_, *bw*, *E*_a_, and *f*_lipid_ will also change the value for *K*_CSTD_ and thus cause a parallel shift of the CSTD function. However, as stated above, the slope of the CSTD function is solely determined by the rate constant of the intake trend, *k*_dec_. Figure 2A also shows that chemicals with slower elimination show larger differences in concentrations measured in individuals of different ages that may be included in CSD. Hence, the slower the elimination kinetics of a chemical, the more important it is to include only empirical CSD with consistent age structure in the time trend analysis.

The relationship of body burden and age is explicitly described by Equation 9, which is illustrated in Figure 2B. As shown in the Supplemental Material (doi:10.1289/ehp.0900648.S1), the relationship between concentration and age is only linear if *k*_elim_ = *k*_dec_; otherwise, the relationship is described by either a convex or concave curve, as has been reported in earlier modeling studies (Alcock et al. 2000; Moser and Mclachlan 2002).

### Model application: a case study of DDT (dichloro diphenyl trichloroethane)

Besides providing an analytical model to clarify the interpretation of the CSTD-based half-life, the second goal of this study is to employ the model to obtain information on human elimination kinetics from CSTD. In particular, we estimated human elimination half-lives of *p*,*p*′-DDT and *p*,*p*′-dichlorodiphenyldichloroethylene (*p*,*p*′-DDE) by applying Equation 7 to CSTD from Sweden (Glynn et al. 2007). We present a five-step procedure, graphically illustrated in Figure 3, that has been implemented in an MS Excel spreadsheet (Microsoft Corporation, Redmond, WA, USA) that is available for download (ETH Zurich, Safety and Environmental Technology Group 2009).

- Step 1: Collect and assemble CSTD, including only individuals whose life history (or at least the major part of it) falls in the postban period [i.e., after 1970 for DDT in Sweden (Hayes 1969)].
- Step 2: Estimate *k*_dec_ from the CSTD by log-linear regression.
- Step 3: Use at least one empirical intake estimate, such as data from a total diet study, in combination with the slope *k*_dec_ from step 2 to describe the time trend of the intake as in Equation 1, again on a log-linear scale.
- Step 4: Extrapolate the function *I*(*t*) from step 3 to the daily intake at *t*_0_, *I*_0_.
- Step 5: Estimate the human elimination rate constant, *k*_elim_, by fitting Equation 7 to the empirical CSTD. Because *k*_dec_ and *I*_0_ have been estimated from the slope of the CSTD and the total diet study in steps 2 and 4, *k*_elim_ is the only fitting parameter in Equation 7.

In principle, *k*_dec_ could be derived from total diet studies rather than from CSTD. However, comprehensive sets of CSTD will become available from biomonitoring programs such as the National Health and Nutrition Examination Survey (NHANES) [Centers for Disease Control and Prevention (CDC) 2009] and the effectiveness evaluation of the Stockholm Convention, which will provide a consistent basis for estimating *k*_dec_ of various chemicals in many parts of the world. In addition, at least a few, ideally several, total diet studies for the same population groups are needed to provide information on *I*_0_; if these studies also yield an estimate of *k*_dec_, this can be used to cross-check the CSTD-based estimate.

Figure 3 illustrates the steps of this procedure with DDT and DDE data from Sweden. Step 1 yields *t*_1/2_^dec^ values of 6.3 years and 8.8 years for *p*,*p*′-DDT and *p*,*p*′-DDE, respectively. We took the intake estimates needed for steps 3 and 4 (open and solid circles in Figure 3A) from two Swedish market-based studies from 1999 (Darnerud et al. 2006) and 2005 (Ankarberg et al. 2006). Because only the sum of DDTs (∑DDT) and *p*,*p*′-DDE were reported explicitly, we estimated *p*,*p*′-DDT intakes as 25% of *p*,*p*′-DDE intakes according to findings from two dietary-intake studies from Canada (Rawn et al. 2004) and Greenland from the same time (Deutch et al. 2006). To assess the plausibility of the intake trends for *p*,*p*′-DDE and *p*,*p*′-DDT extrapolated in steps 3 and 4 (solid and dashed lines in Figure 3A), we collected additional empirical intake estimates that were available only for ∑DDT (Vaz 1995). To compare them with the model results, we calculated the sum of the modeled intake curves for *p*,*p*′-DDE and *p*,*p*′-DDT (dotted line in Figure 3A). Modeled and empirical intake trends of ∑DDT are in good agreement. Modeled intake is slightly below the empirical ∑DDT data possibly because modeled ∑DDT includes only *p*,*p*′-DDE and *p*,*p*′-DDT, whereas empirical ∑DDT also includes dichlorodiphenyl dichloroethane (DDD) and *o*,*p*′-isomers.

Using the five-step procedure, we obtained estimates of the human elimination half-lives, *t*_1/2_^elim^, of 6.2 years for *p*,*p*′-DDE and 2.2 years for *p*,*p*′-DDT. Our estimate for *p*,*p*′-DDE is slightly lower than estimates from sequential measurements in individuals (i.e., LD). Wolff et al. (2000) obtained a median elimination half-life of 8.6 years. For *p*,*p*′-DDT Morgan and Roan (1972) derived an elimination half-life of 1.25 years from measurements in three individuals who had received high doses of 5–20 mg/day. This compares well with our estimate of 2.2 years for individuals exposed to background levels. A slightly faster elimination in the highly dosed individuals is expected because of the concentration dependency of the elimination half-life (Leung et al. 2007). We conducted a second case study using data from the United Kingdom [see Supplemental Material, Table S3 (doi:10.1289/ehp.0900648.S1)]. Elimination half-lives of 7.6 years and 2.1 years for *p*,*p*′-DDE and *p*,*p*′-DDT, respectively, are in very good agreement with the estimates from the Swedish data.

From the most general equation of our PK framework, Equation 5, we have also derived an explicit equation for the body burden measured in CSD as a function of age. This equation, Equation 9, predicts body burdens of chemicals with relatively short elimination half-lives to be less dependent on age. This is consistent with empirical data for DDT, DDE, and different polychlorinated biphenyls (PCBs) (Greve and Vanzoonen 1990; Thomas et al. 2006). For a more detailed description of the properties of Equation 9, see Supplemental Material, Figure S1 and Equation S26 (doi:10.1289/ehp.0900648.S1).

## Discussion

Our analysis provides a transparent interpretation of the half-lives derived from exponential fitting of CSTD. We show that in the postban phase these half-lives reflect a mechanistically defined quantity—the decline of intake by the population—and are independent of human elimination kinetics. To identify CSTD-based half-life estimates from a postban phase unambiguously, we suggest they be referred to as “population exposure half-life.”

In cases where individuals included in the CSTD have spent a significant fraction of their lifetime in a preban period, declining trends may be observed in the CSTD that can also be reasonably fitted with an exponential function (Craan and Haines 1998; Norén and Meironyté 2000). However, in such cases the half-life derived from the empirical fit does not reflect a mechanistically clearly defined quantity. Rather, it reflects influences of the intake trend, which in this case also includes preban intake, the elimination half-life, and the characteristic age of the sampled population [see Supplemental Material, Figure S2 (doi:10.1289/ehp.0900648.S1)]. Under any exposure conditions, CSTD-derived half-lives are specific to the characteristics of the sampled population and the exposure trend, and not individual humans. Therefore, terms including “human” should be avoided when referring to half-lives derived from CSTD.

For a postban period, our analysis clarifies the relationships among *a*) CSTD-based half-life, *b*) time trend of intake, and *c*) the human elimination half-life. In this case, the trend in CSTD directly reflects the trend in exposure; therefore, exponential fitting of CSTD implies the assumption that intake is declining exponentially. For DDT, this is supported by total diet studies presented in Figure 3 and elsewhere (Morisawa et al. 2002). Empirical evidence shows that the assumption of first-order declining intake is also reasonable for other chemicals such as dioxins and PCBs (De Mul et al. 2008). Furthermore, exponential fitting implicitly assumes that the sets of CSD used to derive the CSTD have been obtained from similar groups of individuals. This implicit assumption allows us to model CSTD using a one-compartment PK model that represents individuals with the same average characteristics but born in different years. As a consequence, the solutions to the PK model in Figure 1A do not provide information about interindividual differences, nor do they account for complete lifetime exposure profiles.

Physiologic factors are implemented as constant values in the model, that is, as independent of age. This simplification makes it possible to solve the model analytically; it is supported by two findings. First, background intake and body weight are generally assumed to be proportional in adults (i.e., intake is expressed in units of nanograms per kilogram of body weight per day). Under this assumption, a one-compartment model parameterized for average adults, as presented in Equation 2, is equivalent to a one-compartment model parameterized with age-dependent body weight and background intake proportional to body weight [see Supplemental Material (doi:10.1289/ehp.0900648.S1)]. Second, large changes in daily intake, as they occur in early life years (e.g., due to breast-feeding), also have large effects on the body burden (Moser and McLachlan 2002). However, these concentration peaks level out no later than 20 years of age (Kreuzer et al. 1997; Verner et al. 2008). In other words, predictions of concentrations in average adults as they are represented in CSTD are not strongly influenced by differences in body burdens occurring during early life years due to different breast-feeding regimes (Lorber 2002). If required, the conceptual framework developed here could also be set up with age-dependent physiologic parameters and more general intake functions. In such cases, numerical integration of the model will be necessary.

### Scope of the model framework

Our modeling framework can also be applied to cases that are different from the DDT example. First, the intake function, *I*(*t*), can represent any intake pathway, not only food. In addition, when the model is solved numerically, *I*(*t*) can have any shape [see Supplemental Material, Figure S2 (doi:10.1289/ehp.0900648.S1)]. This means that the model framework is not limited to postban cases. Second, for chemicals stored in parts of the body other than lipids, *bw* × *f*_lipid_ in Equation 2 can be replaced by a description of the actual storage medium, such as the volume of distribution for perfluorinated chemicals. Finally, application of the framework is not limited to persistent chemicals with slow elimination. However, for chemicals that are eliminated quickly, *k*_elim_ can be more readily derived from LD collected from single individuals (Koch and Angerer 2007). Our modeling framework is therefore most powerful when applied to chemicals with slow elimination kinetics such as DDT for two reasons. First, LD studies aiming to estimate *k*_elim_ of slowly eliminating chemicals are difficult to perform because subjects must be tracked over several years, and fasting to eliminate background exposure is not feasible. Second, as demonstrated in Figure 2A, CSTD of slowly eliminating chemicals can only be described by a multi-individual PK model as presented here.

### Model application and performance

For many persistent chemicals, information about human elimination half-lives is scarce (Wolff 1999), and available data exhibit a high degree of uncertainty. For instance, a review of PCB elimination half-lives found reported values ranging from < 1 year to virtually infinity even within a specific congener group (Shirai and Kissel 1996). Different methods have been applied to estimate human elimination half-lives. A common method is to use sequential measurements in individuals who experienced high exposure for a limited time period (e.g., occupational exposure, accident). High-exposure cases are chosen in LD studies to mitigate the problem of ongoing background exposure (Grandjean et al. 2008). However, half-lives derived from such cases may be not representative for the general population because the rate of elimination has been shown to depend, to some extent, on the absolute level of body burden (Leung et al. 2007). An alternative approach to the problem of confounding through ongoing exposure is to account for it in the PK model used to estimate the elimination half-life. Such an expression has been presented for a constant intake (steady-state situation) (Shirai and Kissel 1996). However, because intake often changes over time, integration of the actual intake trend (rather than assuming steady state) would be desirable, but information about intake of measured individuals is rarely available. Here we have shown how time trend information present in CSTD can be combined with information from total-diet studies to determine the intake trend and the whole body elimination half-life for the general adult population. To our knowledge, our method represents the first approach that uses CSTD to estimate human elimination half-lives. The further advantage is that CSTD are available in many countries and represent a broad empirical basis, whereas LD-inferred half-lives are often based on very few (often only two) measurement points (Phillips 1989) with a limited number of subjects available. In two DDT case studies, we employed the model to estimate human elimination half-lives for *p*,*p*′-DDE and *p*,*p*′-DDT using intake estimates and CSTD from Sweden and the United Kingdom as empirical data. Results obtained from both CSTD sets are consistent with each other and with estimates from LD.

### Data quality and availability

The CSTD from Sweden presented in Figure 3 represent only primiparous women with a median age of 28.8 years from the same region (Uppsala County). Hence, they exhibit a relatively high level of consistency. This is because the recurrent measurements had been planned prospectively aiming to establish time trends. In contrast, most studies in the literature that present CSTD-based half-lives have been assembled retrospectively. In many cases only summary statistics are reported, which makes it difficult to compare different sets of CSD. As a result, confounding effects from different age structures and other differences between the studies may occur. In particular, an inconsistent age structure would introduce large uncertainties in CSTD-derived half-lives of slowly eliminated chemicals like POPs.

Therefore, in the future, cross-sectional studies designed to evaluate time trends of POPs in populations should be conducted in such a way that they yield consistent sets of CSTD. Measuring levels in blood and human milk every year in sufficiently large groups of young adults that meet clearly defined physiologic and lifestyle criteria is a particularly effective monitoring strategy. Preferably, cohorts of young adults should be monitored because they meet postban conditions first. Total diet studies, which are already performed in many countries on a regular basis, should be coordinated with these biomonitoring programs to ensure optimal use of resources.

## Conclusions

The present study demonstrates that CSTD used in combination with exposure data can provide quantitative information about human exposure trends and elimination kinetics that has not been exploited so far. We have demonstrated that, under postban conditions, the CSTD-based half-life is equivalent to the population exposure half-life and that the problem of scarcity and uncertainty in estimates of human elimination kinetics of POPs can be reduced by using CSTD in combination with exposure data to derive estimates that represent the adult population under background exposure conditions. Accurate estimates of exposure trends and human elimination kinetics are only possible when the model is applied to consistent CSTD; the acquisition of consistent CSTD requires prospective design of the monitoring programs. Programs such as NHANES and the forthcoming effectiveness evaluation of the Stockholm Convention offer opportunities to apply the conceptual framework presented here to improve the interpretation of biomonitoring data.
